# Hyaluronic Acid-Palmitate Nanoparticle Delivery of Carbonic Anhydrase Inhibitors Impairs Growth and Early Metabolism in *Escherichia coli* Through β- and γ-Carbonic Anhydrase-Associated Processes

**DOI:** 10.3390/ijms27020621

**Published:** 2026-01-07

**Authors:** Viviana De Luca, Valentina Verdoliva, Claudiu T. Supuran, Stefania De Luca, Clemente Capasso

**Affiliations:** 1 Department of Biology, Agriculture and Food Sciences, National Research Council (CNR), Institute of Biosciences and Bioresources, Via P. Castellino, 111, 80131 Naples, Italy; viviana.deluca@ibbr.cnr.it; 2 Department of Environmental, Biological and Pharmaceutical Sciences and Technologies, National Research Council (CNR), Institute of Crystallography, Via Vivaldi, 43, 81100 Caserta, Italy; valentina.verdoliva@cnr.it; 3 Neurofarba Department, Section of Pharmaceutical Sciences, University of Florence, Via Ugo Schiff 6, Sesto Fiorentino, 50019 Florence, Italy; claudiu.supuran@unifi.it; 4 Department of Biomedical Sciences, Institute of Biostructures and Bioimaging, National Research Council (CNR), Via P. Castellino, 111, 80131 Naples, Italy

**Keywords:** carbonic anhydrase, *Escherichia coli*, hyaluronic acid-palmitate nanoparticles, carbonic anhydrase inhibitors, bacterial growth inhibition, energy metabolism

## Abstract

Bacterial carbonic anhydrases (CAs) are essential for intracellular pH regulation, bicarbonate homeostasis, and energy metabolism, making them attractive antimicrobial targets. Here, building on evidence that acetazolamide (AZA) delivered via hyaluronic acid–palmitate (HA-PA) nanocarriers impairs *Escherichia coli* growth and its glucose uptake, we investigated the physiological roles of β- and γ-class CAs using sulphonamide inhibitors with distinct selectivity encapsulated in HA-PA nanomicelles to ensure intracellular delivery. AZA, a potent dual β/γ-CA inhibitor, ethoxzolamide (EZA), a selective β-CA inhibitor, and hydrochlorothiazide (HCT), a weaker inhibitor of both classes, were tested for effects on bacterial physiology. The nanoparticles reduced growth in a dose- and class-dependent manner, with AZA exerting the strongest activity, EZA intermediate inhibition, and HCT only modest effects at higher concentrations. Early metabolic responses assessed via intracellular ATP after three hours of exposure revealed an unexpected and reproducible ATP increase for all inhibitors relative to untreated cells, suggesting reduced ATP consumption in bicarbonate-dependent pathways. These findings provide indirect yet compelling evidence that β- and γ-class CAs influence bacterial energy homeostasis and support the rationale for CA inhibition as an antimicrobial strategy, while highlighting HA-PA carriers as effective systems for delivering CA inhibitors intracellularly and enhancing their functional activity in bacterial cells.

## 1. Introduction

Bacterial carbonic anhydrases (CAs, EC 4.2.1.1) are metalloenzymes that catalyse the reversible hydration of carbon dioxide to bicarbonate and protons, a reaction central to intracellular pH regulation, bicarbonate homeostasis, and overall energy metabolism [1,2,3,4]. In prokaryotes, these enzymes enable adaptation to fluctuating environmental CO_2_ and support efficient central metabolic processes [5,6,7,8]. *Escherichia coli* expresses two main CA classes, β and γ, each encompassing multiple isoforms that contribute to physiological homeostasis [9,10]. Among these, the β-CA isoform CynT2 has been identified as essential for growth under atmospheric CO_2_, highlighting its physiological relevance and making it a prime target for studying the consequences of selective CA inhibition [5,11]. The rise of multidrug-resistant bacteria poses a growing threat to global public health, undermining the efficacy of conventional antibiotics and increasing morbidity and mortality [12,13]. Traditional antimicrobials act by interfering with cell wall biosynthesis, protein translation, nucleic acid replication, or membrane integrity [14,15,16,17]. In contrast, targeting essential metabolic enzymes such as CAs offers a complementary approach [18,19,20]. By disrupting intracellular bicarbonate supply and pH balance, CA inhibition can impair several biosynthetic pathways (mainly involving carboxylation reactions), reduce proliferation, and diminish virulence without relying on classical antibiotic mechanisms [4,21,22]. Previous work from our group demonstrated that acetazolamide (AZA), a well-characterized CA inhibitor (CAI), can suppress *E. coli* growth and metabolic activity when delivered via hyaluronic acid–palmitate (HA-PA) nanocarriers [23,24,25,26]. In this context, substantial efforts have been made to develop drug delivery systems aimed at ensuring the efficient transport of therapeutic agents to their target sites, largely due to the poor diffusion of hydrophobic drugs across the outer membrane. The amphiphilic HA–PA system effectively addresses this challenge by enabling efficient drug encapsulation, enhancing intracellular uptake, and protecting the drug from premature degradation, thereby achieving higher effective concentrations within the bacterial cytoplasm [23,27,28,29]. These results validated CAs as putative antimicrobial targets and highlighted the advantages of nanocarrier-mediated delivery for enhancing inhibitor efficacy [30,31,32,33,34]. We demonstrated that encapsulated AZA modulated glucose consumption in *E. coli*, indicating an impact on bacterial metabolism. These findings suggested that the HA-palmitate nanoparticle not only enhanced intracellular delivery and antibacterial efficacy of AZA but also effectively modulated CA-dependent metabolic pathways [23]. These metabolic changes are consistent with intracellular CA engagement and support the functional relevance of CA inhibition under our experimental conditions. Despite these insights, the contributions of individual CA classes to bacterial growth and metabolism remained unclear. To address this, we employed two additional CAIs with differential class selectivity: ethoxzolamide (EZA), a potent β-CA inhibitor, and hydrochlorothiazide (HCT), a weaker inhibitor of both β- and γ-CAs [11,35]. By using these inhibitors within the HA-PA nanocarrier system, we aimed to link class-specific CA inhibition to early metabolic responses, providing mechanistic insight into how β- and γ-CAs contribute to bacterial growth and homeostasis. Comparison of inhibitors with distinct selectivity profiles further allowed us to delineate the relative roles of β- and γ-CAs in these processes and to establish a functional relationship between CA activity and metabolic regulation, providing a conceptual framework that may guide the future rational design of selective CA-targeted antimicrobials. This strategy may contribute to the development of next-generation antibacterial approaches capable of circumventing the limitations of conventional antibiotics.

## 2. Results and Discussion

**Scheme 1:**
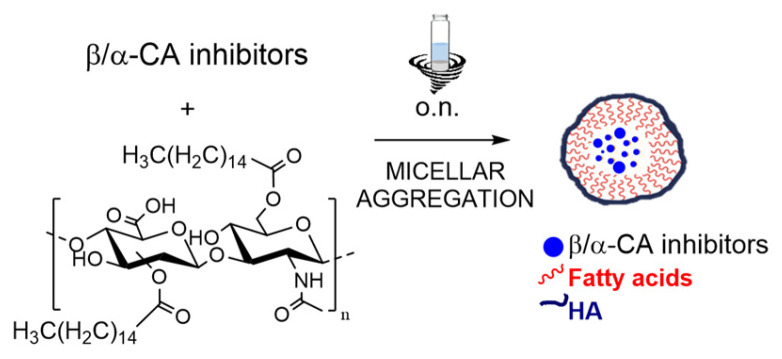
Encapsulation of CAIs into HA–palmitate micelles.

The synthesis of HA–palmitic acid conjugate was performed according to a previously developed green chemistry approach [36,37,38,39]. The procedure is a solvent-free protocol and involves a stepwise process carried out in a single reaction vessel. Briefly, activation of the palmitic acid carboxyl groups is first performed, followed by their esterification with hyaluronic acid (HA) (Scheme 1).

The polysaccharide conjugate was used at concentrations higher than the average critical aggregation concentration (CAC ≈ 0.3 mg/mL) [40], since the CAC is not uniquely defined for synthetic nanocarriers and depends on the degree of hyaluronan functionalization. The well-established emulsion evaporation method was employed to obtain HA–palmitate aggregates that simultaneously encapsulate CAs. Briefly, CAs were dissolved in an organic solvent (chloroform), while HA–palmitate was dissolved in an aqueous NaCl solution (0.9% *w*/*v*). The two solutions were then mixed and maintained under continuous shaking at room temperature overnight. This process allowed the progressive evaporation of the broken-down organic phase, leading to the formation of HA–palmitate aggregates dispersed within the aqueous phase.

**Figure 1:**
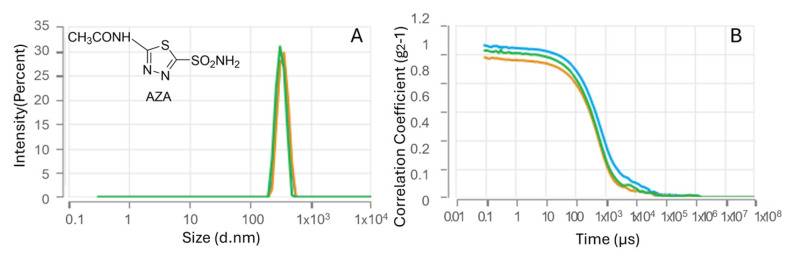
Nanoparticle size analysis (**A**) and DLS intensity autocorrelation data (**B**) for AZA–HA–palmitate formulations.

The characterization of the resulting nanoparticles was subsequently performed by dynamic light scattering (DLS). The AZA-loaded HA–palmitate nanoparticles showed a mean hydrodynamic diameter of 467.7 ± 61.96 nm (Figure 1). Hydrodynamic diameters measured by dynamic light scattering are reported as intensity-weighted size distributions.

**Figure 2:**
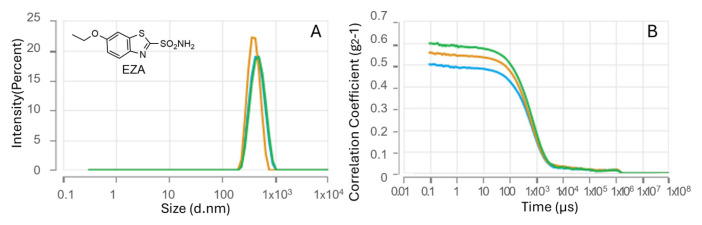
Nanoparticle size analysis (**A**) and DLS intensity autocorrelation data (**B**) for EZA–HA–palmitate formulations.

The EZA-loaded HA–palmitate nanoparticles showed a mean hydrodynamic diameter of 502.4 ± 23.14 nm (Figure 2).

**Figure 3:**
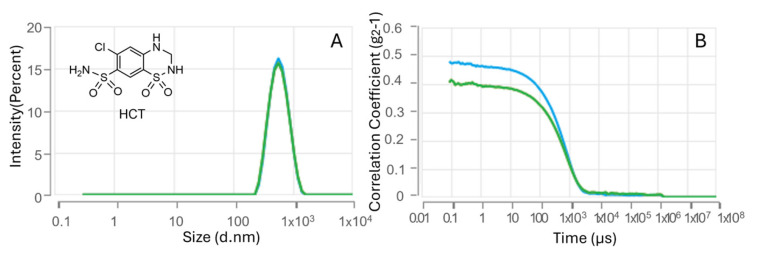
Nanoparticle size analysis (**A**) and DLS intensity autocorrelation data (**B**) for HCT–HA–palmitate formulations.

The HCT-loaded HA–palmitate nanoparticles showed a mean hydrodynamic diameter of 480.1 ± 8.067 nm (Figure 3).

While the relatively large hydrodynamic diameters observed for HA–palmitate nanoparticles (~470–500 nm), which were comparable across AZA-, EZA-, and HCT-loaded formulations, may influence uptake efficiency and intracellular delivery kinetics, uptake was not directly quantified in this study. Nevertheless, the biological effects that will be described in the following sections (growth modulation, ATP accumulation, and MIC profiles) indicate that nanoparticle-mediated delivery was functionally sufficient under the tested conditions; particle size should therefore be considered primarily in the context of future optimization rather than as a limitation of the present results.

Drug entrapment efficiency was evaluated by UV spectroscopy by quantifying the encapsulated drug released from HA–palmitate micelles over a 6-day period at room temperature in phosphate buffer (0.1 M, pH 7.4). Drug concentration was determined by measuring absorbance at 264 nm for AZA, 298 nm for EZA and 270 nm for HCT, respectively. The optimal CAs concentration encapsulated in the micellar systems was 13.2 µg/mL for AZA, 11.9 µg/mL for EZA and 14.9 µg/mL for HCT.

**Figure 4:**
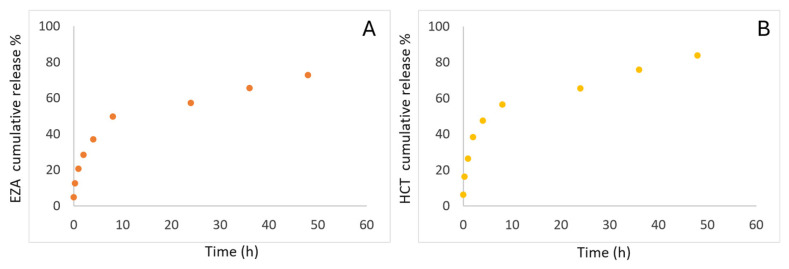
In vitro EZA (**A**) and HCT release study (**B**).

This experiment also enabled the evaluation of the release behavior of CAs (AZA, EZA, HCT) from HA–palmitate nanoparticles over 72 h (performed in triplicate). A progressive drug release was observed, reaching saturation after 24 h. As shown in Figure 4, the percentage of drug released is plotted as a function of time for a representative experiment. Approximately 87.8% for EZA and 89.4% for HCT of the drug was released after 72 h (Figure 4).

### 2.1. Effects of Nanoparticle-Delivered CAIs

#### 2.1.1. Bacterial Growth

**Figure 5:**
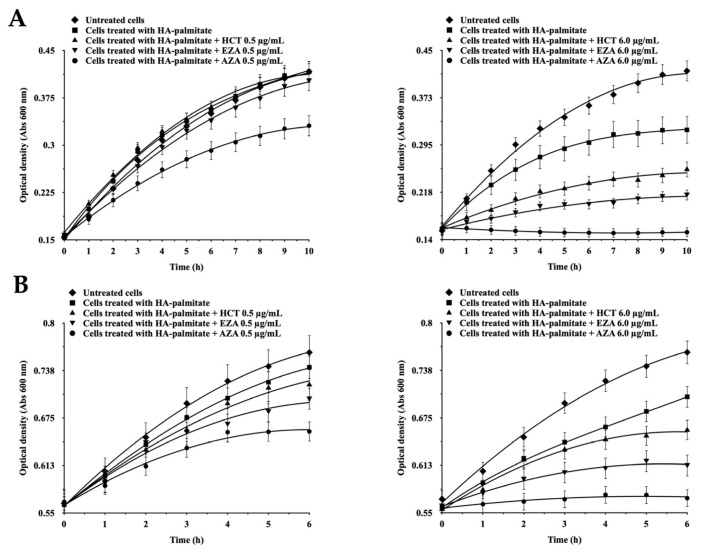
Effects of HA–palmitate nanoparticles loaded with classical sulphonamide CA inhibitors on *E. coli* growth. (**A**) Nanoparticles added at the time of inoculation. (**B**) Nanoparticles added during exponential growth (OD_600_ = 0.5–0.6). Within each panel, left graphs correspond to 0.5 µg/mL and right graphs to 6.0 µg/mL. Symbols indicate untreated cells, HA–palmitate alone, or HA–palmitate loaded with HCT, EZA, or AZA, as specified. Growth was monitored as optical density at 600 nm. Data are means of triplicate measurements; variability was within 3–5%.

Figure 5 illustrates the effects of hyaluronic acid–palmitate (HA-PA) nanoparticles delivering CAIs on *E. coli* growth and metabolism. The inhibitors selected, AZA, EZA, and HCT, are classical sulphonamides with well-characterized inhibitory activity against bacterial CAs. AZA is a potent dual inhibitor targeting both β- and γ-class CAs (K_I_ = 227 nM and 248 nM, respectively); EZA is relatively more selective for β-CA (K_I_ = 557 nM), but not for γ-CA (K_I_ = 5538 nM); and HCT is a weaker inhibitor of both isoforms (K_I_ in the micromolar range) [11,35]. These differences in potency and class selectivity provide a useful framework for interpreting how inhibition of distinct CA classes may translate into physiological outcomes. When bacterial growth was monitored under treatment with 0.5 and 6.0 µg/mL of each compound encapsulated in HA-PA nanoparticles, administered either at inoculation or during exponential growth, clear differences emerged (Figure 5A,B).

AZA consistently produced the strongest inhibitory effect on proliferation, in line with its activity against both β- and γ-class enzymes encoded in the *E. coli* genome. EZA exerted an intermediate effect, consistent with specific targeting of EcoCAβ, while HCT produced minimal inhibition at low concentrations but measurable effects at 6.0 µg/mL, suggesting that its weaker CA activity may only become relevant at higher doses, or that additional CA-independent pathways may contribute. These results reinforce the functional importance of EcoCAβ in sustaining bacterial growth, with EcoCAγ likely providing a complementary but non-redundant role. While these phenotypes are consistent with CA inhibition, they should be interpreted as indirect evidence of target engagement. We cannot exclude that additional factors, such as other sulphonamide-sensitive targets, nanoparticle-associated stress responses, or a broader slowing of metabolism, may contribute to the observed growth modulation.

#### 2.1.2. Intracellular ATP Assay

To probe the metabolic consequences of CA inhibition more directly, intracellular ATP levels (reported as counts, relative luminescence units [RLU]) were measured after 3 h of treatment. This time point was deliberately chosen to capture early enzymatic effects in actively dividing cells, thus minimizing confounding influences from stationary phase, nutrient depletion, or cell death. Importantly, ATP levels were used here as a readout of altered ATP utilization rather than ATP production, with intracellular ATP accumulation interpreted as a marker of reduced anabolic demand (metabolic blockade), not increased bioenergetic efficiency.

As summarized in Table 1, in untreated controls at inoculation, ATP counts reached 763.3 ± 69.5 RLU, whereas cells treated with 0.5 µg/mL of AZA, EZA, or HCT exhibited substantially elevated ATP values of 3806.6 ± 699.5, 2914.6 ± 609.9, and 2499.0 ± 282.1 RLU, respectively (corresponding to 4.9×, 3.8×, and 3.2× increases relative to untreated cells). These marked increases do not reflect enhanced ATP generation; rather, they indicate reduced ATP consumption in the context of impaired biosynthetic flux. When nanoparticles were instead added to cultures already in the exponential growth phase (OD_600_ 0.5–0.6), ATP accumulation was even more pronounced: untreated controls displayed 35,046.6 ± 1681.4 RLU, while treatment with AZA, EZA, or HCT resulted in 475,912.6 ± 22,511.6 (13.5×), 382,203.3 ± 17,946.6 (10.8×), and 150,098.3 ± 5611.2 RLU (4.3×), respectively. The increase in intracellular ATP following CA inhibition is consistent with reduced ATP consumption; however, direct measurements of pathway fluxes were not performed. Accordingly, the ATP and growth phenotypes described here represent supportive functional readouts rather than direct biochemical proof of intracellular CA engagement. In bacteria, CAs maintain intracellular CO_2_/HCO_3_^−^ homeostasis, fuelling numerous anabolic pathways, including nucleotide, fatty acid, and amino acid biosynthesis, which are highly energy-intensive intermediates. When CA activity is suppressed, bicarbonate availability may become limiting, potentially reducing flux through carboxylation-dependent pathways and consequently decreasing bacterial ATP demand. This leads to its intracellular accumulation, which is particularly evident in cells already in the exponential phase, when anabolic activity and energy turnover are at their peak. Because pathway-level measurements (e.g., metabolite profiling or flux analysis) were not performed, these pathway-level effects should be regarded as mechanistic hypotheses supported by indirect phenotypic evidence rather than as demonstrated causal relationships. Although all three inhibitors induced ATP increases at both inoculation and exponential phase treatment, their effects on biomass accumulation differed. AZA produced the strongest reduction in proliferation, consistent with its broader inhibition spectrum, whereas EZA and HCT, while still elevating ATP, caused weaker effects on growth (Figure 5). This inverse relationship between ATP accumulation and bacterial biomass indicates that stronger growth inhibition is associated with higher intracellular ATP retention, supporting the interpretation that ATP buildup serves as an early marker of metabolic blockade rather than enhanced energy generation. The magnitude of ATP accumulation followed the order AZA > EZA > HCT, directly mirroring their relative inhibitory strength on bacterial growth. Conceptually, CA inhibition is expected to reduce bicarbonate-dependent anabolic throughput, lowering ATP demand for biosynthesis and resulting in intracellular ATP retention, particularly in metabolically active cells. In the literature, it has been reported that when metabolic pathways are blocked (e.g., by many antimicrobial agents, toxins or excess adenine), ATP accumulates, not being consumed for biosynthesis and growth [41,42,43,44]. The fold-change values reported in Table 1 further illustrate that ATP dynamics are more sensitive to CA inhibition than biomass accumulation, emphasizing their utility as an early readout of enzymatic perturbation. This observation is consistent with our previous demonstration that AZA-loaded HA-palmitate nanoparticles modulated glucose consumption in *E. coli*, further supporting that nanoparticle-delivered CAIs interfere with intracellular, CA-dependent metabolic fluxes. The convergence of reduced glucose consumption (previous work), ATP accumulation (this study), and growth inhibition (both studies) is consistent with intracellular CA-dependent metabolic perturbation. Taken together, these findings indicate that nanoparticle-mediated CA inhibition perturbs bacterial physiology in two interconnected ways: (i) it reduces proliferation in an inhibitor specific manner, and (ii) it is associated with altered ATP homeostasis consistent with reduced consumption. The dissociation between biomass accumulation and ATP dynamics, as highlighted by the fold-increase values, provides mechanistic insight into how EcoCAβ sustains growth and metabolic flux, while the enhanced ATP response in exponential phase cultures underscores the importance of the cellular metabolic state in modulating inhibitor efficacy. These findings are based on early metabolic and growth phenotypes, which provide indirect but coherent functional indications of CA involvement, rather than direct evidence of enzymatic target engagement. Although a detailed delineation of the intracellular pathways affected by CA inhibition lies beyond the scope of this study, the consistent pattern observed across class-selective inhibitors and metabolic readouts supports a unified mechanistic interpretation. Overall, the data are consistent with a significant contribution of β- and γ-class CAs to bacterial energy homeostasis, although off-target responses and indirect effects cannot be excluded.

### 2.2. Minimum Inhibitory Concentration (MIC)

To further quantify the antibacterial activity of HA-PA-encapsulated CAIs, we determined the minimum inhibitory concentration (MIC) and attempted to determine the minimum bactericidal concentration (MBC) for AZA, EZA, and HCT against *E. coli*. Consistent with the standard broth microdilution method in accordance with CLSI guidelines, MICs were defined at 24 h and MBCs assessed by subculture. As summarized in Table 2, AZA exhibited the lowest MIC (4.0 µg/mL), followed by EZA (8.0 µg/mL) and HCT (10.0 µg/mL). Because MIC values reflect a 24 h growth-inhibition threshold rather than target-specific potency, even CA inhibitors with lower intrinsic activity or distinct selectivity patterns, such as EZA and HCT, may converge to MIC values of similar magnitude. To determine whether the observed growth inhibition represented bactericidal or non-bactericidal effects, MBC assays were subsequently performed.

### 2.3. Minimum Bactericidal Concentration (MBC)

To distinguish between bactericidal and non-bactericidal effects, we next determined the minimum bactericidal concentrations (MBCs) of the HA-PA-encapsulated CA inhibitors. Viable colonies were recovered from all wells upon subculture onto drug-free agar, including those corresponding to the highest concentration tested (10.0 µg/mL), and therefore no measurable MBC values could be determined within the tested range (4.0–10.0 µg/mL) (Table 2). These results indicate that inhibition of bacterial CAs by AZA, EZA, and HCT produces a predominantly bacteriostatic, rather than bactericidal, effect under the conditions of our assay. This outcome is fully consistent with a metabolism-targeting mode of action, in which cellular growth is suppressed without rapid cell killing. Thus, whether bactericidal effects emerge at higher multiples of MIC or longer exposure remains untested.

The absence of measurable MBC values for AZA, EZA, and HCT indicates that the growth inhibition produced by these HA-PA-encapsulated CA inhibitors under our assay conditions does not involve detectable bactericidal activity. In this context, CA inhibition may be more appropriately envisioned as a strategy for growth modulation or metabolic weakening of bacterial cells, or as an adjuvant approach that enhances the efficacy of other antimicrobial agents, rather than as a standalone bactericidal therapy. This outcome is compatible with the metabolic mode of action expected for primary sulphonamide CA inhibitors, which modulate enzymatic pathways rather than inducing rapid cell killing. Whether prolonged exposure, higher multiples of MIC, or combination therapies could shift this response toward bactericidal outcomes remains an open question beyond the scope of the present study.

## 3. Materials and Methods

### 3.1. Compounds

HA sodium salt from *Streptococcus equi* (15–30 kDa molecular weight), palmitic acid, DIC, K_2_CO_3_, CHCl_3_, AZA, EZA, HCT and Mueller–Hinton (MH) broth were purchased from Sigma-Aldrich (St. Louis, MO, USA). Spectrum™ Labs Spectra/Por™ 3 3.5 kDa MWCO Standard RC Dry Dialysis Kits were purchased from Thermo Fisher Scientific (Waltham, MA, USA); *E. coli* DH5α were purchased from Agilent Technologies (Santa Clara, CA, USA). The INNOVA 42 incubator was obtained from Eppendorf (Hamburg, Germany).

### 3.2. Preparation and Size Characterization of HA–Palmitate Micelles Encapsulating β/γ-CA Inhibitors

HA–palmitate nanoparticles incorporating the selected β/γ-CA inhibitors were prepared using an evaporation approach [45]. Briefly, the inhibitors were first solubilized in CHCl_3_ (1 mL) to obtain solutions at a final concentration of 50 µg/mL, while HA–palmitate (3 mg) was dispersed separately in 0.9% (*w*/*v*) NaCl aqueous solution (3 mL). The two phases were emulsified with brief sonication, followed by gentle stirring overnight at room temperature to allow the self-assembly of the polymeric nanoparticles. Evaporation of the organic phase yielded an aqueous dispersion of HA–palmitate nanoparticles encapsulating the β/γ-CA inhibitors. The aqueous phase was subsequently centrifuged at 13,000× *g* for 10 min, and unincorporated, insoluble CAIs were eliminated by size-exclusion chromatography using a Sephadex G50 column (GE Healthcare, Uppsala, Sweden).

The purified nanoparticles were finally analysed by Dynamic Light Scattering (DLS) using a Zetasizer Pro instrument (Malvern Panalytical, Almelo, The Netherlands). The experimental settings were as follows: temperature 25.0 °C; disposable microcuvettes (40–45 µL).

### 3.3. In Vitro Drug Release Investigation

The release profiles of EZA and HCT from HA-palmitate micelles were evaluated using a dialysis-based method. Micellar formulation (2 mg), containing known amount of encapsulated drug, was suspended in 0.9% (*w*/*v*) NaCl aqueous solution (2 mL) and transferred into a dialysis membrane bag (cut-off 3.5 kDa). The dialysis bag was subsequently immersed in a beaker containing 20 mL of phosphate buffer (0.1 M, pH 7.4) and kept under continuous magnetic stirring for 72 h to evaluate drug release. At specified time intervals, aliquots of the release medium (2 mL) were collected for analysis and replaced with an equal volume of fresh buffer to preserve constant volume and sink conditions.

The amount of released drug was quantified by UV-vis measurement at 298 nm for EZA and 270 nm for HCT.

The cumulative drug release was monitored over time until no further significant increase in drug concentration was observed. Under these conditions, complete drug release was assumed to be achieved at 170 h, which was therefore considered as the maximum release time (t_max_).

The percentage of drug release was calculated according to the following equation:

$$Drug\;release\;\left(\%\right)=\frac{Amount\;of\;drug\;released\;at\;time\;t}{Total\;amount\;of\;drug\;release\;}\;\times \;100$$

The total drug released at t_max_ = 170 h was considered as the total drug content encapsulated in the micelles. This corresponds to a maximum concentration of 13.2 µg/mL for AZA (Abs_264_ = 0.74), 11.9 µg/mL for EZA (Abs_298_ = 0.585) and 14.9 µg/mL for HCT (Abs_270_ = 0.701), representing 100% of the encapsulated drug. This method was therefore also used to determine the total amount of drug encapsulated within the micelles. The percentages of EZA and HCT released over 72 h, corresponding to 87.8% and 89.4%.

UV-vis spectra were recorded on a Jasco V-730 spectrophotometer (ETCS-761, Easton, MD, USA) over 220–350 nm using 500 μL quartz cuvettes, with blank correction applied. Measurements were performed in continuous scan mode with the following parameters: scan rate 200 nm/min, data interval 0.2 nm, response time 0.24 s, and spectral bandwidth 1.0 nm.

### 3.4. Bacterial Growth Assays

To assess the effects of HA-PA-encapsulated CAIs (AZA, EZA, and HCT) on *E. coli* proliferation, 200 µL of bacterial suspension (10^5^ cells) was dispensed into 96-well plates. HA-PA nanoparticles loaded with each inhibitor were added either at inoculation or during the exponential growth phase (OD_600_ = 0.5–0.6), using two concentrations of the encapsulated inhibitor: 0.5 µg/mL and 6 µg/mL. Cultures treated at OD_600_ 0.5–0.6 were monitored for 6 h following inhibitor addition, a time window chosen to capture early effects on proliferation before stationary-phase entry or significant cell death. Optical density at 600 nm (OD_600_) was measured hourly using a microplate reader (Tecan SPARK Multimode Microplate Reader, Männedorf, Switzerland). Untreated cultures and cultures exposed to unloaded HA-PA nanoparticles served as controls. All experiments were conducted in biological triplicate to ensure reproducibility.

### 3.5. ATP Assay

Intracellular ATP levels in *E. coli* were measured using the ATP Bioluminescence Assay Kit CLS II (Roche, Basel, Switzerland) according to the manufacturer’s guidelines [46,47]. The preparation of cultures and the timing of inhibitor addition matched those used in the bacterial growth assay, except that a single inhibitor concentration (0.5 µg/mL of encapsulated AZA, EZA, or HCT) was employed. This dose was selected because it reliably elicits early metabolic effects while preserving comparable cell viability across treatments. Untreated cells were collected at time zero and processed identically to the treated samples, providing baseline intracellular ATP levels. Treated cultures were harvested after 3 h of exposure to the inhibitor-loaded HA-PA nanoparticles. A 3 h incubation was selected because it captures early metabolic responses to CA inhibition while cultures remain in the exponential phase, thereby avoiding confounding effects from growth slowdown, nutrient limitation, or cell death. After this incubation period, an equal number of cells was used for ATP determination. Bacterial suspensions from each condition were standardized to 10^5^ cells/mL, ensuring that identical cell numbers were processed per sample. Nine volumes of boiling extraction buffer (100 mM Tris, 4 mM EDTA, pH 7.75) were then added, and samples were incubated at 100 °C for 2 min to achieve complete lysis and ATP release. Lysates were centrifuged at 1000× *g* for 60 s and the supernatants were kept on ice until analysis. Aliquots (50 µL) of samples or standards were transferred to white microplate wells, mixed with an equal volume of luciferase reagent (1:1), and luminescence was measured with a 1 s integration time. Background luminescence was subtracted, and ATP concentrations were calculated from the standard curve. Because identical cell numbers were processed for each condition, ATP values directly reflect differences in intracellular ATP content per cell. Unloaded HA-PA nanoparticles were tested to exclude assay interference. All measurements were performed in biological triplicate, with each sample assayed in technical triplicate, and values are reported as mean ± SD.

### 3.6. Minimum Inhibitory Concentration (MIC) and Minimum Bactericidal Concentration (MBC)

The antimicrobial activity of HA-PA-encapsulated inhibitors (AZA, EZA, and HCT) was evaluated using the standard broth microdilution method in accordance with CLSI guidelines [48]. Briefly, *E. coli* cultures were adjusted to 10^5^ CFU and exposed to a series of twofold serial dilutions starting from 10 µg/mL, resulting in the following concentrations: 10.0, 8.0, 4.0, 2.0, 1.0, 0.50, and 0.25 µg/mL. This approach allowed a systematic evaluation of dose-dependent effects while maintaining experimental reproducibility. The MIC was defined as the lowest concentration at which no visible bacterial growth was observed after 24 h of incubation at 37 °C (OD_600_ < 0.05 relative to untreated control) [49]. To determine the MBC defined as the lowest concentration resulting in a ≥99.9% reduction in viable bacterial counts relative to the initial inoculum, 10 µL aliquots from wells showing no visible growth were plated on LB agar and incubated for 24 h at 37 °C [50]. All measurements were conducted in biological triplicate, with results expressed as mean ± SD. This method provides a clear and reproducible quantification of the antibacterial efficacy of each inhibitor, linking observed growth inhibition to precise, measurable concentrations.

## 4. Conclusions

This study demonstrates that HA-palmitate nanoparticles provide an effective strategy for delivering CAIs to *E. coli*, modulating both bacterial growth and early metabolic activity. In this regard, the green chemistry approach to designing drug-loaded nanoparticles is widely encouraged. This goal can be considered achieved based on the obtained results. Overall, the experimental data consistently indicate that nanoparticle-mediated delivery of carbonic anhydrase inhibitors results in a clear inhibition of bacterial growth and early alterations of intracellular energy homeostasis. The combined analysis of growth curves, intracellular ATP levels, and susceptibility assays provides a coherent descriptive summary of the main experimental findings. Among the inhibitors tested, AZA produced the strongest effects, consistent with potent dual inhibition of β- and γ-class CAs. EZA induced intermediate responses, reflecting selective β-CA inhibition, whereas HCT exerted measurable effects only at higher concentrations, suggesting potential off-target contributions. Notably, intracellular ATP levels increased within three hours of treatment, suggesting an altered energy homeostasis under the exposure to the CAIs as well as that metabolic perturbations precede overt growth bacterial inhibition. The concordant rank order AZA > EZA > HCT in both growth and ATP assays, together with the amplified response in exponential-phase cultures, strongly supports CA target engagement and an early-onset metabolic mechanism. In agreement with our previous findings, showing that AZA-loaded HA-palmitate nanoparticles disrupt glucose consumption, these results provide additional indirect evidence of intracellular CA inhibition. HA-PA–encapsulated CAIs showed MICs but no MBC at ≤8.0 μg/mL (24 h), indicating bacteriostatic activity under the tested conditions only. This pharmacodynamic behaviour is consistent with the metabolic mode of action of CA inhibition and with the non-essentiality of CA-dependent CO_2_/bicarbonate interconversion for *E. coli* survival under nutrient-rich, aerobic conditions. Collectively, these observations provide strong mechanistic evidence for the central role of EcoCAβ in sustaining metabolic fluxes and underscore the complexity of drug–bacteria interactions, where inhibitor potency, CA-class selectivity, and off-target effects converge. Overall, combining selective CA inhibition with HA-palmitate nanoparticle delivery offers a promising strategy for developing metabolism-targeted antimicrobials. By exploiting the fundamental contribution of CAs to microbial physiology, this approach could complement traditional antibiotics and support the rational design of non-bactericidal yet growth-modulating therapies with potential applications against multidrug-resistant pathogens.

## Figures and Tables

**Table 1:** Intracellular ATP levels in *E. coli* following treatment with HA–palmitate nanoparticles loaded with CA inhibitors. ATP levels are reported as relative luminescence units (RLU) measured after 3 h of treatment, either at inoculation or during exponential growth (OD600 = 0.5–0.6).

Treatment (0.5 µg/mL)	ATP at Inoculation Phase (RLU)	Fold vs. Control	ATP at Exponential Phase (RLU)	Fold vs. Control
Control	763.3 ± 69.5		35,046.6 ± 1681.4	
AZA	3806.6 ± 699.5	4.9×	475,912.6 ± 22,511.6	13.5×
EZA	2914.6 ± 609.9	3.8×	382,203.3 ± 17,946.6	10.8×
HCT	2499.0 ± 282.1	3.2×	150,098.3 ± 5611.2	4.3×

**Table 2:** MIC and MBC of HA-PA-encapsulated CAIs against *E. coli*.

CA Inhibitor	MIC (µg/mL)	* MBC (µg/mL)
AZA	4.0	ND
EZA	8.0	ND
HCT	10.0	ND

* ND, no bactericidal activity was detected at the highest concentration tested; MBC values could not be determined within the tested range.

## Data Availability

All data generated or analyzed during this study are included in this published article. These comprise data from nanoparticle preparation and characterization (dynamic light scattering, drug loading and release profiles), spectrophotometric measurements, microplate-based bacterial growth and ATP assays, and MIC/MBC determinations. No additional datasets were generated.
